# Supplementary material for: Addressing cultural and knowledge barriers to enable preclinical sex inclusive research
Source: eLife. 2025 Dec 10;14:RP106545. doi: 10.7554/eLife.106545 (PMC12695025; doi:10.7554/eLife.106545)
Supplement: Supplementary file 11. — The data and SAS code which can reproduce the analysis of intention for study 1 and study 2. [file elife-106545-supp11.pdf]

Intention data and SAS analysis code

The following provides the data and SAS code used to analyze study 1 and study 2 intention data. This information can be used for data and analysis transparency. Further, the information below can be cut and pasted directly into SAS to reproduce the analysis.

Data and analysis code 1: data and analysis code for figure 1

```
DATA Survey_data_no_select_questions2; INPUT  InfluencePlanningExp2 StatsCourses2
&$11. Familiar_with_Factorials2 Prev_Sex_Incorporation2 Exposure_TRT &$17.
AvgAttitude AvgBehavControl AvgSocNorm What_is_your_age_ Gender2 &$ Geography2
&$9. How_many_years_have_you_worked_w Type_of_work2 &$19. Education2 &$
AvgIntent_Box_Cox_3_3_2; Lines;
4  1-2 courses  4  25  Interest in topic  7  5.66666666666667  4.66666666666667
32  Woman  Asia  10  Basic biol invest  Doctoral  3.17416428286284
3  1-2 courses  3  50  Interest in topic  7  4.66666666666667  6  29  Man  Asia  3
Drug devel/Lead opt  MS  3.54700262719911
3  No courses  2  0  Interest in topic  5.5  3.66666666666667  4.66666666666667
29  Woman  Australia  8  Basic biol invest  MS  1.61227392145414
5  1-2 courses  2  0  Interest in topic  7  4.33333333333333  2.33333333333333  26
Woman  Australia  6  Basic biol invest  Other  1.88434514569953
4  1-2 courses  3  25  Interest in topic  7  4.33333333333333  6.33333333333333
28  Woman  Australia  5  Discovery  Doctoral  3.93999439555356
3  1-2 courses  2  0  Interest in topic  5  2.66666666666667  3.66666666666667  27
Man  Australia  3  Basic biol invest  Other  0.916980792827043
5  No courses  2  100  Interest in topic  5.5  5.66666666666667  5.33333333333333
27  Woman  Europe  3  Basic biol invest  MS  4.35313958792618
3  1-2 courses  2  0  Interest in topic  6  4.66666666666667  3  51  Man  Asia  25
Drug devel/Lead opt  Doctoral  2.17656979396309
4  1-2 courses  2  25  Interest in topic  7  3.33333333333333  4  27  Man  Europe
7  Basic biol invest  MS  2.82147936254475
5  No courses  3  75  Interest in topic  7  5.66666666666667  6  53  Woman  Other
20  Basic biol invest  Doctoral  4.35313958792618
3  No courses  2  50  Interest in topic  4  4.66666666666667  5  40  Woman  Asia
15  Discovery  Doctoral  1.61227392145414
2  1-2 courses  2  0  Interest in topic  7  2.66666666666667  2  25  Woman  Europe
4  Other  Doctoral  2.48894786624483
3  1-2 courses  1  50  Interest in topic  7  5.66666666666667  6  56  Woman  Other
10  Drug devel/Lead opt  Doctoral  3.17416428286284
3  >2 courses  1  25  Interest in topic  6  3.33333333333333  2.33333333333333  62
Woman  Europe  35  Basic biol invest  Doctoral  1.1285917450179
5  1-2 courses  4  75  Interest in topic  7  3.33333333333333  5.66666666666667
43  Woman  Europe  13  Basic biol invest  Doctoral  3.54700262719911
5  1-2 courses  2  25  Interest in topic  5.75  4  1.66666666666667  32  Man
Europe  6  Basic biol invest  Doctoral  1.61227392145414
5  1-2 courses  4  75  Interest in topic  7  4  5  36  Woman  Europe  4  Basic
biol invest  Doctoral  3.93999439555356
5  >2 courses  4  50  Interest in topic  6.75  5  4.66666666666667  35  Woman
Europe  15  Basic biol invest  Doctoral  4.35313958792618
4  >2 courses  3  25  Interest in topic  7  4.33333333333333  3  27  Woman  Europe
5  Discovery  MS  3.93999439555356
5  1-2 courses  4  100  Interest in topic  7  4.66666666666667  5.66666666666667
57  Woman  Europe  35  Basic biol invest  Doctoral  4.35313958792618
5  1-2 courses  2  25  Interest in topic  6.75  3  5.33333333333333  30  Man
Europe  7  Basic biol invest  Doctoral  1.61227392145414
```

5 1-2 courses 4 100 Interest in topic 7 6.66666666666667 5.33333333333333
49 Woman Europe 20 Discovery Other 4.35313958792618
5 1-2 courses 5 75 Interest in topic 7 5 6.66666666666667 35 Woman
Europe 12 Basic biol invest Doctoral 4.35313958792618
3 1-2 courses 3 100 Interest in topic 7 4.66666666666667 5 28 Woman
Europe 3 Basic biol invest MS 2.82147936254475
4 No courses 2 100 Interest in topic 7 6.33333333333333 6 30 Woman
Europe 5 Basic biol invest Doctoral 3.17416428286284
4 1-2 courses 2 75 Interest in topic 7 4.33333333333333 2.66666666666667
44 Woman Europe 5 Drug devel/Lead opt Doctoral 2.82147936254475
3 No courses 2 0 Interest in topic 6.5 2.33333333333333 2 30 Man Europe
7 Discovery MS 1.1285917450179
5 >2 courses 2 25 Interest in topic 4 4.66666666666667 4.33333333333333 35
Woman Asia 12 Basic biol invest Doctoral 1.36035612122693
4 No courses 3 100 Interest in topic 7 6 5.66666666666667 30 Man Europe
8 Basic biol invest Doctoral 3.17416428286284
4 No courses 2 0 Interest in topic 5.25 4.33333333333333 4.33333333333333
29 Man Asia 5 Basic biol invest MS 1.36035612122693
3 1-2 courses 2 0 Interest in topic 7 2 1.66666666666667 24 Woman Europe
2 Basic biol invest MS 2.48894786624483
5 1-2 courses 2 25 Interest in topic 7 5.33333333333333 5.66666666666667
34 Man Europe 13 Basic biol invest Doctoral 3.54700262719911
4 No courses 2 25 Interest in topic 6 4.33333333333333 4 24 Woman Asia
0 Basic biol invest MS 2.17656979396309
5 >2 courses 3 75 Interest in topic 6 5 6 28 Man Asia 5 Basic biol
invest MS 3.17416428286284
4 1-2 courses 2 25 Interest in topic 7 3 5.33333333333333 23 Woman Other
4 Basic biol invest Other 4.35313958792618
5 >2 courses 4 50 Interest in topic 5.75 4.66666666666667 5.33333333333333
44 Man Europe 20 Drug devel/Lead opt Doctoral 2.17656979396309
4 1-2 courses 1 25 Interest in topic 5 4 4 26 Man Europe 3 Drug
devel/Lead opt Other 2.82147936254475
5 No courses 2 100 Interest in topic 7 6 6 24 Woman Europe 1 Basic
biol invest Other 4.35313958792618
3 1-2 courses 3 25 Interest in topic 6 3.66666666666667 5 50 Man Europe
3 Basic biol invest Doctoral 2.48894786624483
3 1-2 courses 1 25 Interest in topic 4.75 4 4 25 Woman Europe 2 Basic
biol invest MS 1.88434514569953
4 No courses 4 75 Interest in topic 6 4.33333333333333 5 35 Man
Australia 3 Drug devel/Lead opt MS 2.82147936254475
4 No courses 4 25 Interest in topic 6 3.33333333333333 4.66666666666667 65
Man Europe 38 Basic biol invest Doctoral 1.36035612122693
2 1-2 courses 3 0 Gen Pop 6.5 4.66666666666667 2.66666666666667 22 Man
Europe 4 Basic biol invest Other 1.88434514569953
2 1-2 courses 3 25 Gen Pop 6.75 4 3.66666666666667 21 Woman Europe 1
Other Other 3.54700262719911
5 >2 courses 3 50 Gen Pop 7 4.66666666666667 7 53 Man Australia 32
Discovery Doctoral 4.35313958792618
5 No courses 3 75 Gen Pop 6.25 3.33333333333333 6 54 Woman Australia 35
Discovery Doctoral 3.93999439555356
5 >2 courses 3 25 Gen Pop 7 7 2.33333333333333 77 Man Other 53 Basic
biol invest Doctoral 1.36035612122693
5 1-2 courses 3 50 Gen Pop 7 5 5.66666666666667 54 Woman Australia 25
Basic biol invest Doctoral 3.17416428286284
5 1-2 courses 3 0 Gen Pop 4.25 3.33333333333333 3.33333333333333 27 Man
Asia 2 Basic biol invest MS 2.17656979396309
3 1-2 courses 1 50 Gen Pop 7 4 5 21 Man Europe 1 Basic biol invest
Other 3.93999439555356

4 >2 courses 4 25 Gen Pop 7 4.33333333333333 4.33333333333333 42 Woman
Other 7 Basic biol invest Doctoral 2.17656979396309
5 1-2 courses 2 0 Gen Pop 7 5 1.33333333333333 29 Woman Europe 2 Basic
biol invest Doctoral 1.36035612122693
5 1-2 courses 4 50 Gen Pop 6.5 5.66666666666667 4 51 Man Other 30
Basic biol invest Doctoral 3.54700262719911
5 1-2 courses 4 25 Gen Pop 3.75 2.66666666666667 3 56 Woman Europe 25
Discovery Doctoral 0.272071224245386
3 No courses 3 75 Gen Pop 7 3.66666666666667 6 41 Man Other 3 Drug
devel/Lead opt Doctoral 3.17416428286284
5 >2 courses 3 50 Gen Pop 7 4.33333333333333 3.66666666666667 37 Man
Europe 12 Basic biol invest Doctoral 1.1285917450179
3 1-2 courses 3 100 Gen Pop 7 4 4 24 Woman Other 3 Other MS
2.48894786624483
5 1-2 courses 3 50 Gen Pop 7 6 6.66666666666667 42 Woman Europe 19
Basic biol invest Doctoral 4.35313958792618
5 1-2 courses 2 25 Gen Pop 6.25 3 5.33333333333333 34 Woman Europe 5
Basic biol invest Doctoral 3.17416428286284
3 No courses 2 0 Gen Pop 7 2.33333333333333 5.33333333333333 24 Woman
Europe 1 Basic biol invest MS 2.82147936254475
2 No courses 2 100 Gen Pop 7 4.66666666666667 5.66666666666667 26 Woman
Europe 1 Drug devel/Lead opt MS 1.88434514569953
4 No courses 4 75 Gen Pop 7 4 5.33333333333333 55 Man Europe 30 Basic
biol invest Doctoral 3.17416428286284
4 1-2 courses 2 25 Gen Pop 5.75 4.33333333333333 3.33333333333333 75 Man
Europe 52 Discovery Doctoral 1.61227392145414
3 1-2 courses 2 25 Gen Pop 7 3.33333333333333 4.33333333333333 23 Woman
Europe 0.5 Basic biol invest Other 3.93999439555356
5 No courses 2 50 Gen Pop 7 5 4.66666666666667 32 Woman Europe 8 Basic
biol invest Doctoral 3.17416428286284
5 1-2 courses 5 75 Gen Pop 7 6 5.66666666666667 56 Woman Australia 35
Discovery Doctoral 4.35313958792618
4 1-2 courses 3 0 Gen Pop 2.5 4.33333333333333 6.33333333333333 30 Woman
Europe 3 Basic biol invest MS 0.554219160499861
4 No courses 2 25 Gen Pop 6.25 3 4.66666666666667 37 Woman Other 10
Basic biol invest Doctoral 2.17656979396309
4 No courses 2 75 Gen Pop 7 3.66666666666667 5.33333333333333 74 Woman
Other 40 Other Doctoral 3.17416428286284
3 1-2 courses 1 0 Gen Pop 7 4.66666666666667 6 38 Woman Other 10 Basic
biol invest MS 3.17416428286284
3 No courses 2 75 Gen Pop 6 5 5.33333333333333 51 Woman Australia 30
Basic biol invest Doctoral 3.17416428286284
5 1-2 courses 4 100 Gen Pop 6 5 6.33333333333333 25 Woman Europe 8
Drug devel/Lead opt MS 4.35313958792618
5 1-2 courses 5 75 Gen Pop 7 2.66666666666667 4 45 Woman Europe 20
Basic biol invest Doctoral 4.35313958792618
4 No courses 4 25 Gen Pop 7 6 3.33333333333333 55 Man Asia 35 Basic
biol invest Doctoral 3.17416428286284
4 >2 courses 4 100 Gen Pop 6.5 5.33333333333333 4 40 Man Europe 10
Other Doctoral 2.48894786624483
5 1-2 courses 3 75 Gen Pop 7 3.66666666666667 6.33333333333333 39 Man
Europe 15 Basic biol invest Doctoral 3.17416428286284
5 1-2 courses 1 100 Gen Pop 5 3.66666666666667 4.33333333333333 27 Woman
Asia 4 Other Doctoral 1.36035612122693
5 >2 courses 5 75 Interest in topic 7 4.33333333333333 3.33333333333333 56
Man Europe 30 Basic biol invest Doctoral 2.17656979396309
4 No courses 4 50 Interest in topic 7 4 3 41 Woman Australia 8 Basic
biol invest Doctoral 3.17416428286284

```
4  >2 courses  3  75  Interest in topic  7  4.33333333333333  5  38  Other  Europe
15  Basic biol invest  Doctoral  2.17656979396309
4  1-2 courses  4  25  Interest in topic  7  4.33333333333333  5.66666666666667
46  Woman  Europe  23  Basic biol invest  Doctoral  3.17416428286284
4  1-2 courses  4  50  Interest in topic  6  5  4.66666666666667  29  Woman  Asia
7  Basic biol invest  MS  0.272071224245386
5  No courses  2  0  Interest in topic  6.75  4  5.66666666666667  51  Woman
Europe  30  Basic biol invest  Doctoral  4.35313958792618
5  No courses  3  100  Workshop  5.5  6  5.66666666666667  27  Woman  Europe  3
Basic biol invest  MS  4.35313958792618
5  1-2 courses  5  50  Workshop  7  4  7  35  Woman  Europe  12  Basic biol invest
Doctoral  3.54700262719911
5  1-2 courses  4  25  Workshop  7  5  6.66666666666667  52  Man  Australia  30
Discovery  Doctoral  4.35313958792618
2  No courses  4  75  Workshop  7  6.33333333333333  6.66666666666667  57  Man
Europe  25  Basic biol invest  Doctoral  3.93999439555356
4  1-2 courses  2  0  Workshop  6  2.33333333333333  1  26  Woman  Other  6  Drug
devel/Lead opt  MS  2.48894786624483
5  1-2 courses  4  75  Workshop  7  5  5.66666666666667  36  Woman  Europe  5
Basic biol invest  Doctoral  4.35313958792618
4  >2 courses  4  50  Workshop  7  5.33333333333333  6.33333333333333  35  Woman
Europe  15  Basic biol invest  Doctoral  3.93999439555356
4  1-2 courses  3  50  Workshop  7  4  3.33333333333333  58  Woman  Europe  35
Other  Doctoral  3.17416428286284
4  No courses  3  75  Workshop  7  4.66666666666667  6  30  Man  Europe  8  Basic
biol invest  Doctoral  3.93999439555356
4  1-2 courses  3  100  Workshop  6.5  4.66666666666667  4.66666666666667  32
Woman  Asia  7  Other  MS  3.17416428286284
5  1-2 courses  3  100  Workshop  7  5.66666666666667  4.33333333333333  57  Woman
Europe  35  Basic biol invest  Doctoral  4.35313958792618
5  No courses  3  100  Workshop  7  6  6  58  Man  Australia  38  Discovery
Doctoral  4.35313958792618
4  >2 courses  3  50  Workshop  7  6  5  33  Man  Other  12  Basic biol invest
Doctoral  3.17416428286284
5  1-2 courses  3  75  Workshop  6  3.66666666666667  3  52  Man  Asia  30  Drug
devel/Lead opt  MS  3.17416428286284
3  >2 courses  2  50  Workshop  7  5  7  52  Man  Asia  3  Basic biol invest  MS
4.35313958792618
;
RUN;

PROC GLM  DATA=Survey_data_no_select_questions2 ALPHA=0.05;
CLASS StatsCourses2 Exposure_TRT Gender2 Geography2 Type_of_work2 Education2;
MODEL  AvgIntent_Box_Cox_3_3_2 =  AvgAttitude AvgBehavControl AvgSocNorm
Exposure_TRT What_is_your_age_ Gender2 Geography2 How_many_years_have_you_worked_w
Type_of_work2 Education2 StatsCourses2 Familiar_with_Factorials2
Prev_Sex_Incorporation2 InfluencePlanningExp2;
RUN;
```

Data analysis code 2: data and analysis code for figure 2

```
DATA TPB_no_select_analysis20240319; INPUT  ParticipantInitials &$ Pre_Post &$16.
InfluencePlanningExp2 FamilarwithFactorials2 HowOftenIncorpSex2 AvgAttitude
AvgBehavControl AvgSocNorm AvgIntent What_is_your_age_ Gender2 &$; Lines;
MIP  Pre  4  1  25  7  5.33333333333333  4  5.33333333333333  39  Woman
SSS  Pre  4  4  100  5.75  5.66666666666667  6.33333333333333  7  38  Woman
```

CKTL	Pre	1	2	0	7	2.66666666666667	4	5	27	Man
AAA	Pre	4	4	50	7	3.33333333333333	6.33333333333333	6.33333333333333	36	Woman
EAS	Pre	4	3	75	7	4	6	5.33333333333333	36	Man
YD	Pre	3	1	100	7	5	5	7	27	Woman
MSU	Pre	4	2	25	7	3	5.66666666666667	6	42	Man
AJH	Pre	4	4	100	7	6.66666666666667	4.66666666666667	6	55	Man
AC	Pre	4	2	75	7	5	6.33333333333333	7	36	Woman
RBN	Pre	3	2	25	5	2	5.66666666666667	4.66666666666667	46	Woman
AJM	Pre	4	3	100	6.5	5	4	6	42	Woman
AMS	Pre	4	4	25	7	3.66666666666667	6.66666666666667	6	50	Other
JJD	Pre	4	5	75	7	4.66666666666667	7	7	25	Woman
MC	Pre	1	4	100	7	4.33333333333333	5.66666666666667	7	29	Woman
EMJ	Pre	4	2	100	6.75	6	4.66666666666667	6.66666666666667	29	Woman
SFA	Pre	3	5	25	6.5	3	4.33333333333333	4	30	Other
PEE	Pre	3	3	25	7	4.33333333333333	4.66666666666667	5	.	Man
CMG	Pre	4	2	50	7	3.66666666666667	3	4	25	Woman
DDD	Pre	2	2	25	6.25	4	4	6	28	Man
ksk	Pre	3	2	25	5	3.33333333333333	5.66666666666667	5.66666666666667	60	Man
RYX	Pre	3	1	75	7	2	6	6	38	Woman
DRH	Pre	1	2	75	7	3.66666666666667	5.33333333333333	6	27	Man
SMC	Pre	2	2	75	6.5	3	3	2.66666666666667	33	Woman
NNK	Pre	2	1	75	7	2.66666666666667	4.33333333333333	5.33333333333333	35	Woman
GJF	Pre	4	2	100	7	4	7	7	28	Man
OMO	Pre	4	3	100	7	4	5.66666666666667	6.33333333333333	35	Woman
SSS	Post	4	4	100	6.25	6.33333333333333	6.33333333333333	6	38	Other
JWH	Pre	1	3	0	4.75	2	4.33333333333333	4.33333333333333	43	Man
SFA	Post	2	5	0	6.75	3.66666666666667	3.66666666666667	3	30	Other
DRH	Post	1	5	0	7	5.33333333333333	6.33333333333333	6.33333333333333	28	Other
EAS	Post	4	4	0	7	3.66666666666667	7	7	36	Other
AJH	Post	4	4	0	7	6	5.33333333333333	6	.	Other
MIP	Post	4	4	0	7	6	4	6	39	Other
Tw	Post	3	3	0	7	3.66666666666667	5	7	29	Other
EMJ	Post	4	4	0	6.75	6	4.66666666666667	7	29	Other
AC	Post	4	4	0	7	6.33333333333333	7	7	36	Other
AAA	Post	4	5	0	7	3.66666666666667	7	7	36	Other
PEE	Post	3	3	0	7	6	5.66666666666667	5	56	Other
EJS	Post	4	2	0	7	4.33333333333333	4.33333333333333	6	34	Other
MC	Post	2	3	0	7	4	4.33333333333333	7	29	Other
JWH	Post	1	4	0	7	5	6.66666666666667	4.66666666666667	43	Other
CKTL	Post	1	2	0	7	4	4.33333333333333	4.66666666666667	27	Other
AJM	Post	4	4	0	7	5.66666666666667	4.33333333333333	6	42	Other
RYX	Post	3	3	0	7	5	6.33333333333333	7	38	Other
MI	Pre	4	1	25	7	4.33333333333333	4.66666666666667	5.33333333333333	39	Woman
ksk	Post	3	3	0	6.5	4.33333333333333	6	6	60	Other
DDD	Post	1	2	0	7	3.66666666666667	5.33333333333333	7	28	Other
JJD	Post	4	5	0	7	5	6.66666666666667	7	26	Other
CMG	Post	3	4	0	7	3.33333333333333	4	5	25	Other
RBN	Post	3	2	0	7	4	6	5.66666666666667	46	Other
NNK	Post	2	3	0	7	4	4.66666666666667	5.66666666666667	35	Other
OMO	Post	4	4	0	7	4	6	6	35	Other
GJF	Post	4	4	0	7	4.33333333333333	7	7	28	Other
SMC	Post	2	4	0	7	3.33333333333333	2.66666666666667	4	33	Other
ABC	Post	3	2	0	7	4	6.33333333333333	6	30	Other
MSU	Post	4	3	0	7	4	6	6	42	Other

```
;
RUN;

PROC MIXED ASYCOV NOBOUND  DATA=TPB_no_select_analysis20240319 ALPHA=0.05;
CLASS ParticipantInitials Pre_Post Gender2;
MODEL  AvgIntent =  AvgAttitude AvgBehavControl AvgSocNorm Pre_Post
What_is_your_age_ Gender2 InfluencePlanningExp2 FamilarwithFactorials2
HowOftenIncorpSex2/ SOLUTION DDFM=KENWARDROGER;
RANDOM ParticipantInitials / SOLUTION ;
RUN;
```
