# Supplementary material for: Addressing cultural and knowledge barriers to enable preclinical sex inclusive research
Source: eLife. 2025 Dec 10;14:RP106545. doi: 10.7554/eLife.106545 (PMC12695025; doi:10.7554/eLife.106545)
Supplement: Supplementary file 12. — The Rmarkdown output from the analysis of the cumulative knowledge score for study 1. [file elife-106545-supp12.pdf]

Analysis of cumulative knowledge score by treatment group

Natasha Karp

15th November 2023

Study 1

Data Preparation

Import & wrangling:

```
surveydf <- read.csv(here("Survey1_CumulativeKnowledgeMetric.csv"))
surveydf$Group <- factor(surveydf$Group,
                          levels = c("Conference", "Symposium", "Workshop"),
                          labels = c("Baseline", "Interested",
"Intervention"))
summary(as.factor(surveydf$Gender2))

##   Man Other Woman
##    41     1    62

surveydf_2sexes <- subset(surveydf, surveydf$Gender2 != "Other")
levels(as.factor(surveydf_2sexes$Gender2))

## [1] "Man"   "Woman"

head(surveydf_2sexes)

##   ParticipantID AvgAttitude AvgBehavControl AvgSocNorm AvgIntent.BoxCox
Group Gender2 CumulativeKnowledgeScore
## 1            93        5.50        3.666667   4.666667        1.6671796
Interested   Woman                        1
## 2            96        7.00        4.333333   6.333333        4.0741703
Interested   Woman                        1
## 3            97        5.00        2.666667   3.666667        0.9482084
Interested     Man                        0
## 4           102        6.00        4.666667   3.000000        2.2506925
Interested     Man                        3
## 5           105        6.25        4.666667   3.333333        2.5737086
Interested     Man                        1
## 6           109        7.00        5.666667   6.000000        4.5013850
Interested   Woman                        2
```

Visualisation study 1

Visual inspection of data suggests an effect of intervention that is not dependent on gender.

```r
pcPlot <- ggplot(data = surveydf, aes(y = CumulativeKnowledgeScore,x = Group,
col = Group)) + ylab("Cumulative knowledge Score") + xlab("Group")
pcPlot + geom_violin() + theme(axis.text.x = element_text(angle = 45, vjust =
1, hjust = 1))
```

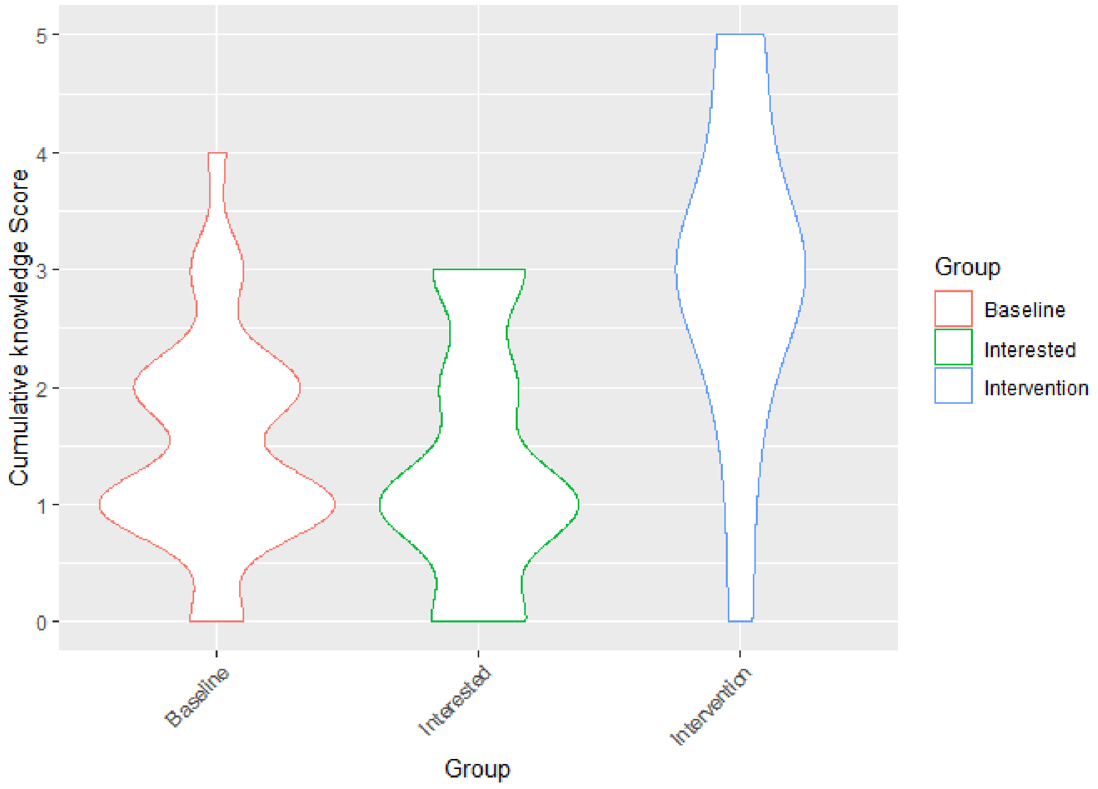


```r
pcPlot <- ggplot(data = surveydf_2sexes, aes(y = CumulativeKnowledgeScore,x =
Group, col = Group)) + ylab("Cumulative knowledge Score") + xlab("Group")
pcPlot + geom_violin() + theme(axis.text.x = element_text(angle = 45, vjust =
1, hjust=1))+ facet_grid(. ~ Gender2)
```

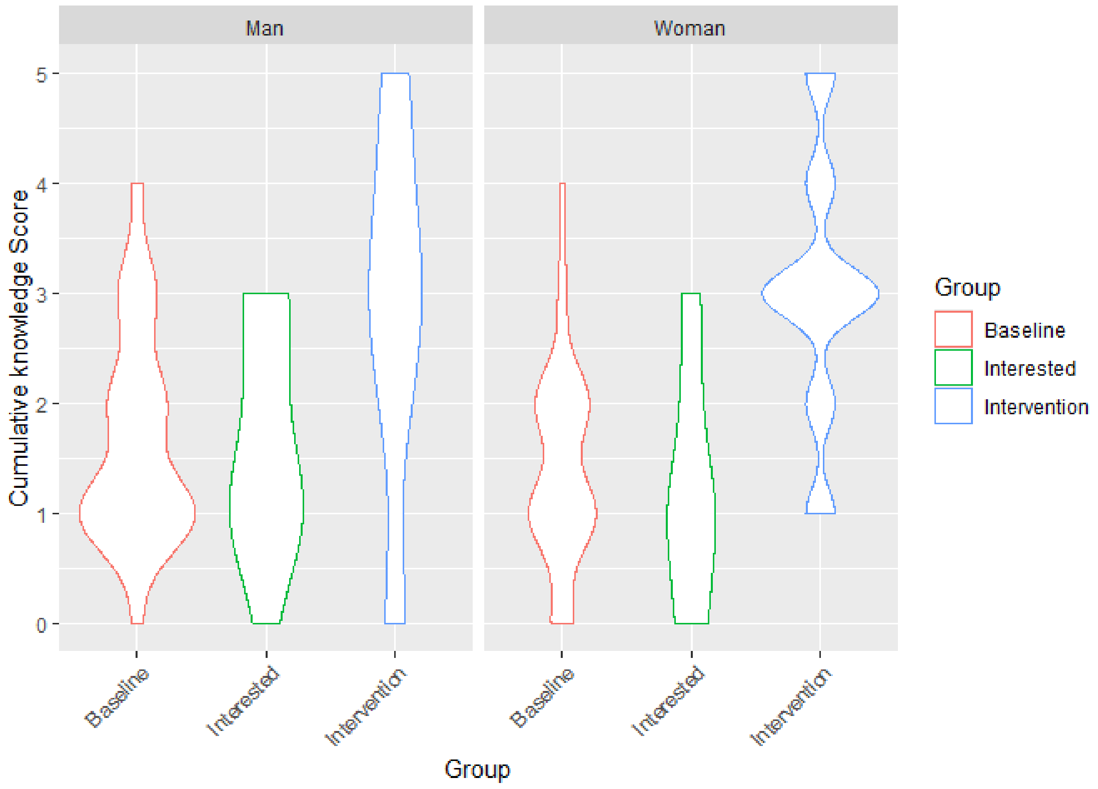

Statistical Analysis Study 1

A poisson regression analysis assessing the role of treatment group in explaining variation in the cumulative knowledge score.

Reference group is the conference

Conclusions:

1. Symposium group is not significantly different to reference
2. Workshop is statistically significantly different with B=0.66342 indicating the expected count is (exp(B))= 1.94 fold larger than the reference group.

```
#Assumptions of a poisson distribution
#Poisson Response the response variable is a count per unit of time or space,
described by a Poisson distribution.
#Independence The observations must be independent of one another.
#Mean=Variance By definition, the mean of a Poisson random variable must be
equal to its variance.
#Linearity The log of the mean rate, log(lambda), must be a linear function
of x.

poisson.model <- glm(CumulativeKnowledgeScore ~ Group, surveydf_2sexes,
family = poisson(link = "log"))
summary(poisson.model)
```

```
##
## Call:
## glm(formula = CumulativeKnowledgeScore ~ Group, family = poisson(link =
"log"),
##     data = surveydf_2sexes)
##
## Coefficients:
##                     Estimate Std. Error z value Pr(>|z|)
## (Intercept)          0.41110    0.10600   3.878 0.000105 ***
## GroupInterested     -0.09872    0.18875  -0.523 0.600943
## GroupIntervention    0.66342    0.18875   3.515 0.000440 ***
## ---
## Signif. codes:  0 '***' 0.001 '**' 0.01 '*' 0.05 '.' 0.1 ' ' 1
##
## (Dispersion parameter for poisson family taken to be 1)
##
##     Null deviance: 91.143  on 102  degrees of freedom
## Residual deviance: 77.614  on 100  degrees of freedom
## AIC: 304.83
##
## Number of Fisher Scoring iterations: 5
```

```
#model checks
# 1: Check whether the model has over-dispersion or under-dispersion. If the
Residual Deviance is greater than the degrees of freedom, then over-
dispersion exists. This means that the estimates are correct, but the
standard errors (standard deviation) are wrong and unaccounted for by the
model.
#the residual deviance < df suggesting over-dispersion is not an issue


#2 goodness fit test testing hypothesis that the model is appropriate - p-
value of Residual deviance goodness-of-fit test
1 - pchisq(deviance(poisson.model), df = poisson.model$df.residual)
```

```
## [1] 0.9526611
```

```
#3 Diagnostic plots
par(mfrow = c(2,3))
plot(poisson.model, which = 1:6)
```

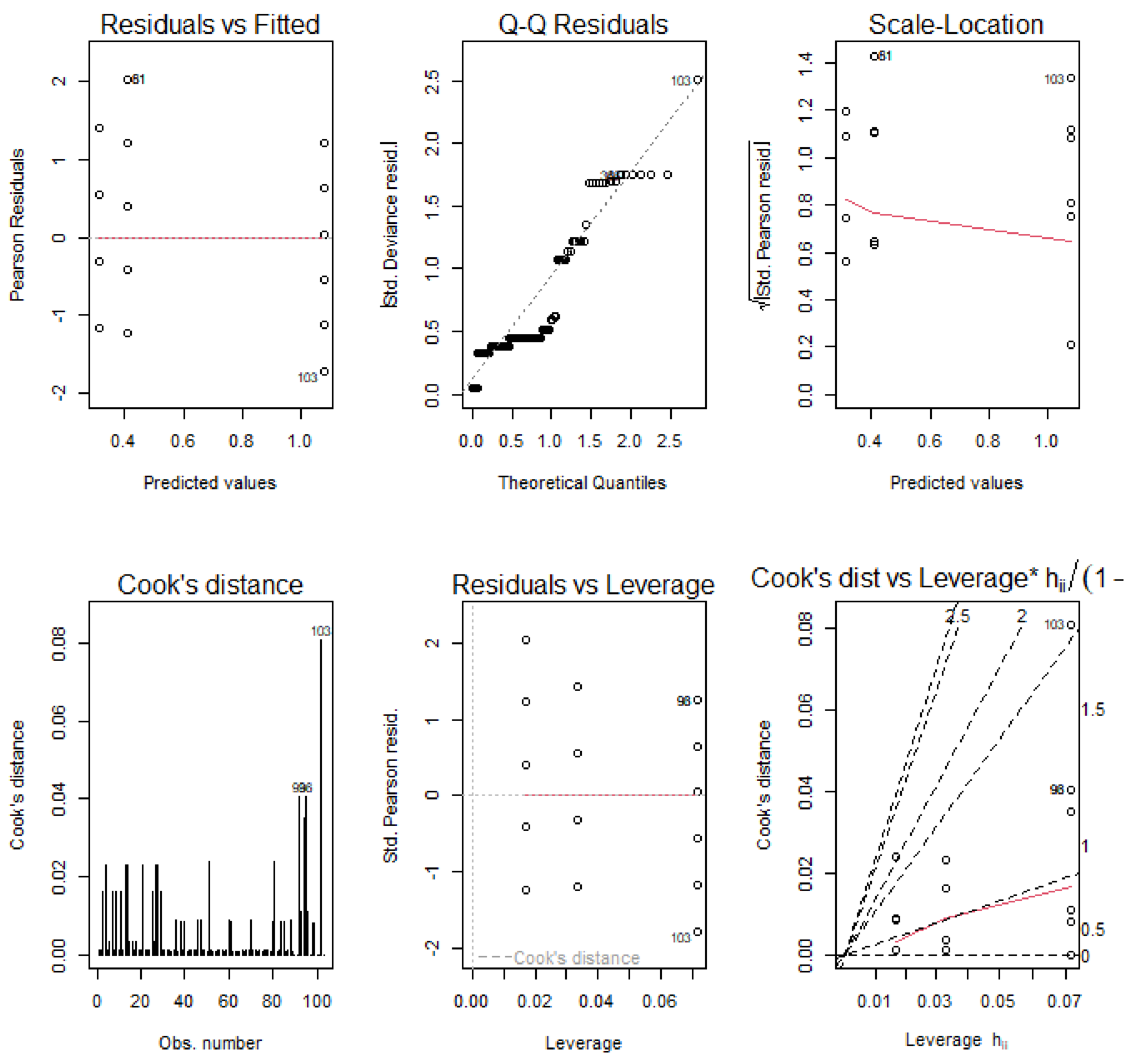


```
#https://www.statology.org/how-to-identify-influential-data-points-using-cooks-distance/#:~:text=A%20general%20rule%20of%20thumb,to%20identify%20influential%20data%20points.
#rule of thumb  - any point with a Cook’s Distance over 4/n (where n is the total number of data points) is considered to be an outlier.
#4/103
#diagnostics looks acceptable

# Calculating the ES as a meaningful value - If Beta > 0, then exp(Beta) > 1, and the expected count is exp(Beta) times larger than when X = 0
exp(0.66342)

## [1] 1.941421

plot_summs(poisson.model, scale = TRUE, exp = TRUE)
```

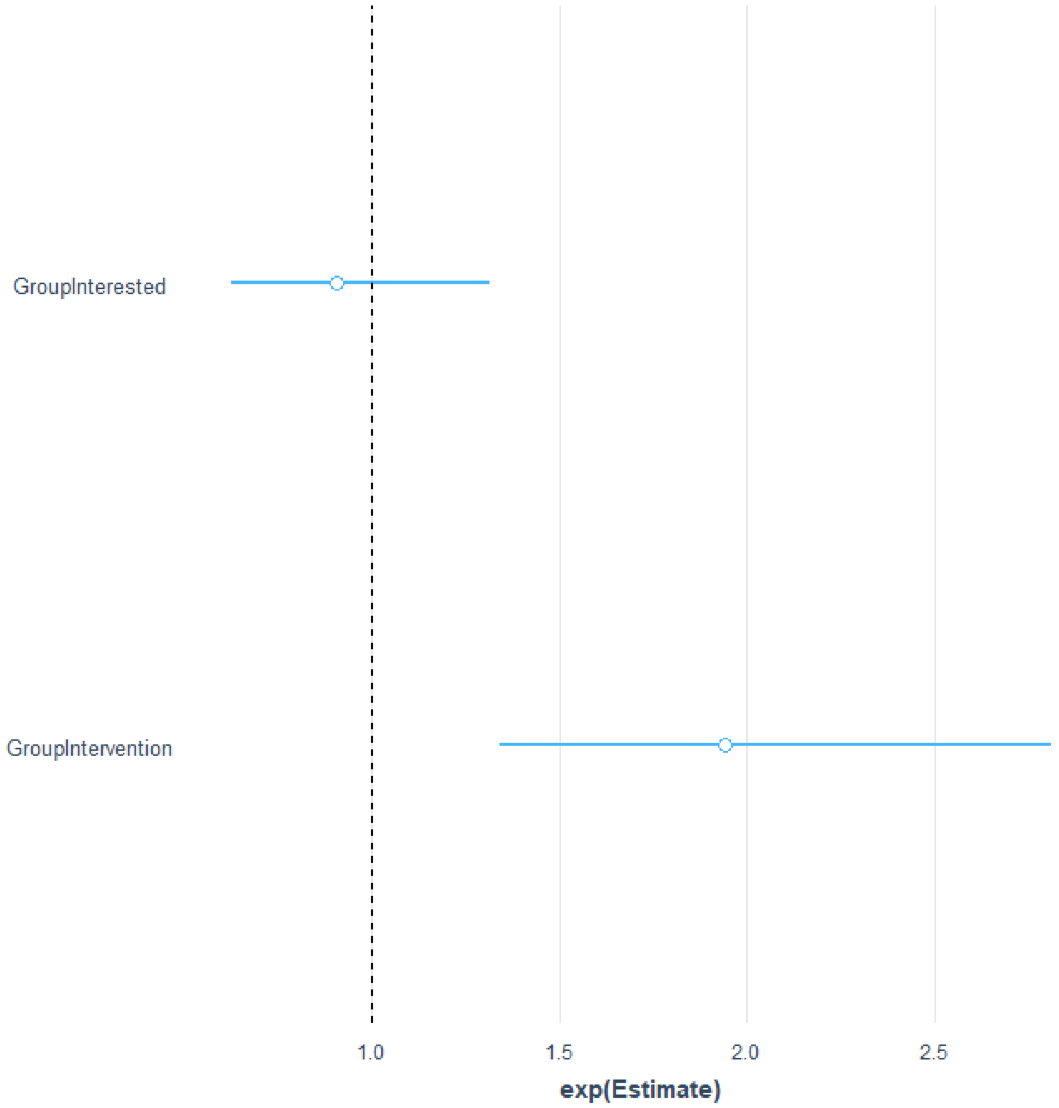

Session Info

```
pander(sessionInfo())
```

R version 4.3.1 (2023-06-16 ucrt)

Platform: x86_64-w64-mingw32/x64 (64-bit)

locale: *LC_COLLATE=English_United Kingdom.utf8*, *LC_CTYPE=English_United Kingdom.utf8*, *LC_MONETARY=English_United Kingdom.utf8*, *LC_NUMERIC=C* and *LC_TIME=English_United Kingdom.utf8*

attached base packages: *stats*, *graphics*, *grDevices*, *utils*, *datasets*, *methods* and *base*

other attached packages: *here(v.1.0.1)*, *emmeans(v.1.10.4)*, *pander(v.0.6.5)*, *jtools(v.2.2.2)* and *ggplot2(v.3.5.1)*

loaded via a namespace (and not attached): *broom.mixed(v.0.2.9.5)*, *future(v.1.34.0)*, *tidyr(v.1.3.0)*, *sandwich(v.3.1-1)*, *utf8(v.1.2.3)*, *generics(v.0.1.3)*, *lattice(v.0.21-8)*, *listenv(v.0.9.0)*, *digest(v.0.6.37)*, *magrittr(v.2.0.3)*, *evaluate(v.1.0.0)*, *grid(v.4.3.1)*, *estimability(v.1.5.1)*, *mvtnorm(v.1.2-2)*, *fastmap(v.1.2.0)*, *rprojroot(v.2.0.3)*, *Matrix(v.1.6-0)*, *backports(v.1.5.0)*, *survival(v.3.5-5)*, *multcomp(v.1.4-26)*, *purrr(v.1.0.1)*, *fansi(v.1.0.4)*, *scales(v.1.3.0)*, *TH.data(v.1.1-2)*, *codetools(v.0.2-20)*, *cli(v.3.6.1)*, *rlang(v.1.1.1)*, *crayon(v.1.5.3)*, *parallelly(v.1.38.0)*, *munsell(v.0.5.0)*, *splines(v.4.3.1)*, *withr(v.2.5.0)*, *yaml(v.2.3.7)*, *parallel(v.4.3.1)*, *tools(v.4.3.1)*, *coda(v.0.19-4.1)*, *dplyr(v.1.1.2)*, *colorspace(v.2.1-0)*, *forcats(v.1.0.0)*, *globals(v.0.16.2)*, *broom(v.1.0.7)*, *vctrs(v.0.6.3)*, *R6(v.2.5.1)*, *zoo(v.1.8-12)*, *lifecycle(v.1.0.3)*, *MASS(v.7.3-60)*, *furrr(v.0.3.1)*, *pkgconfig(v.2.0.3)*, *pillar(v.1.9.0)*, *gtable(v.0.3.3)*, *glue(v.1.6.2)*, *Rcpp(v.1.0.11)*, *xfun(v.0.39)*, *tibble(v.3.2.1)*, *tidyselect(v.1.2.0)*, *highr(v.0.10)*, *rstudioapi(v.0.15.0)*, *knitr(v.1.43)*, *farver(v.2.1.2)*, *xtable(v.1.8-4)*, *nlme(v.3.1-162)*, *htmltools(v.0.5.8.1)*, *rmarkdown(v.2.23)*, *labeling(v.0.4.2)* and *compiler(v.4.3.1)*
